# Assessing the improvement of healthcare workers’ alcohol-based hand rub with the Pulpe'Friction audit

**DOI:** 10.3205/dgkh000603

**Published:** 2025-12-05

**Authors:** Daniel Verbouk, Muriel Péfau, Raymond Nasso, Pierre Parneix, Mélanie Ohanian, Fanny Velardo, Anne-Gaëlle Venier

**Affiliations:** 1 Center for Prevention of Healthcare Associated Infections of Nouvelle Aquitaine, CPias Nouvelle-Aquitaine, Mission nationale MATIS, Bordeaux, France; 2 Center for Prevention of Healthcare Associated Infections of Iles de Guadeloupe, CPias Iles de Guadeloupe, Mission nationale MATIS, Pointe à Pitre, France

**Keywords:** self-reported audit, hand hygiene, patient experience, audit

## Abstract

Hand hygiene is essential to prevent the transmission of pathogens between healthcare workers (HCWs) and patients. Launched in 2019, Pulpe’Friction is a declarative audit that provides a quick assessment of hand hygiene in healthcare facilities. This audit consists of interviewing HCWs and patients. Some facilities performed a second audit after the first one, as well as a sufficient period of implementation of actions. The question was, did these facilities improve in terms of hand hygiene practices and informating the patients?

In two stable cohorts of French medical-facility wards, this study aimed to assess whether there was an improvement in reported and observed alcohol based hand rub (ABHR) practices and in the proportion of patients informed about hand hygiene when two audits were conducted consecutively.

A first cohort of 416 wards from 130 facilities was constituted including 5,521 HCWs interviewed during the first audit and 5,383 HCWs during the second audit. A second cohort of 139 wards from 62 facilities was formed including 2,090 patients interviewed during the first audit and 1,726 patients during the second audit. A significant improvement was observed in self-reported alcohol hand rub practices by HCWs before and after contact with the patient, and after contact with the patient’s immediate environment. Significantly, more patients declared they received information about when to perform hand hygiene during the second audit.

To conclude, the wards in which two audits were performed significantly improved their reported practices. The pulpe’friction method is not only useful to evaluate reported practices and barriers, it also offers a simple way to evaluate the impact of the actions implemented in a ward.

## Abstract (de)

Händehygiene ist unerlässlich, um die Übertragung von Krankheitserregern zwischen medizinischem Personal (MP) und Patienten zu verhindern. Pulpe’Friction wurde 2019 eingeführt und ist ein deklariertes Audit, das eine schnelle Bewertung der Händehygiene in Gesundheitseinrichtungen ermöglicht. Das Audit besteht aus der Befragung von MP und Patienten. Einige Einrichtungen führten nach dem ersten Audit und einer theoretischen Phase der Umsetzung von Maßnahmen ein zweites Audit durch. Die Frage war, haben sich in diesen Einrichtungen die Händehygienepraktiken und die Information der Patienten verbessert?

Das Ziel der Studie bestand darin, an zwei stabilen Kohorten französischer Stationen zu beurteilen, ob es eine Verbesserung der gemeldeten und beobachteten Praktiken des Einsatzes von Alkohol basierten Händedesinfektionsmitteln und des Anteils der Patienten gab, die über Händehygiene informiert wurden, wenn zwei Audits nacheinander durchgeführt wurden.

Es wurde eine erste Kohorte von 416 Stationen aus 130 Einrichtungen zusammengestellt. 5.521 Mitarbeiter*innen wurden während des ersten Audits und 5.383 während des zweiten Audits befragt. Es wurde eine zweite Kohorte von 139 Stationen aus 62 Einrichtungen gebildet (darunter 2.090 Patienten, die während des ersten Audits und 1.726 Patienten, die während des zweiten Audits befragt wurden). Eine signifikante Verbesserung wurde bei den von den Mitarbeiter*innen des Gesundheitswesens angegebenen Praktiken zum Einreiben der Hände vor und nach dem Kontakt mit den Patienten sowie nach dem Kontakt mit der unmittelbaren Umgebung der Patienten beobachtet. Deutlich mehr Patienten gaben an, dass sie beim zweiten Audit Informationen darüber erhalten hätten, wann sie Händehygiene durchführen sollten.

Zusammenfassend lässt sich sagen, dass die Stationen, in denen zwei Audits durchgeführt wurden, ihre gemeldeten Praktiken deutlich verbessert haben. Die Pulpe’friction-Methode eignet sich nicht nur zur Bewertung gemeldeter Praktiken und Hindernisse, sondern bietet auch eine einfache Möglichkeit, die Auswirkungen der auf einer Station durchgeführten Maßnahmen zu bewerten.

## Introduction

Hand hygiene is essential to prevent the transmission of pathogens from healthcare workers (HCWs) to patients and from patients to HCWs [1], [2], [3], [4], [5], [6]. When hands are visibly clean, it is recommended to use alcohol-based hand rub (ABHR) as it is not only more effective microbiologically but also saves time [2], [4], [6]. Different tools exist to assess ABHR, such as Pulpe’Friction audit [7], [8]. Launched in 2019, Pulpe’Friction is a declarative audit that provides a quick assessment of hand hygiene in a ward. This audit consists of a short interview of HCWs and patients. The questionnaire for the HCWs evaluates their ABHR practices and the barriers they face in the field. The questionnaire for the patients evaluates whether they received information about hand hygiene and evaluates their experience of the HCWs’ ABHR practices before a performing care action. 

In France, facilities were encouraged to carry out Pulpe’Friction audits in order to evaluate and implement actions to improve ABHR. It was recommended that after they conducted an audit, they implement actions in a ward. A second Pulpe’Friction audit could be performed to assess the impact of the intervention. 

Until now, no cohort study was performed on Pulpe’Friction databases. Some healthcare facilities used Pulpe’Friction and performed a second audit after they implemented some actions in the ward to fight the barriers they identified with the HCWs. The question was, did these facilities show an improvement in and hygiene practices and the informating the patients?

Thus, the aim of this study was to assess whether there was an improvement in reported and observed ABHR practices and the proportion of patients informed about hand hygiene when two audits were conducted.

## Materials and methods

### Study design

The method of Pulpe’friction audit has already been described in detail in previous papers [7], [8], [9]. An investigator interviewed HCWs and/or patients individually, using specific communication elements to obtain answers as close to reality as possible. Some wards decided to interview only HCWs, some interviewed only patients and some interviewed both. Pulpe’friction gives a collective analysis of the practices declared by the HCWs of a ward and identifies the main barriers for hand rub. The Pulpe’Friction audit offers personalized advice to improve the use of ABHR in wards that performed the audit. When debriefing with the HCWs, improvement actions are decided collectively to improve hand hygiene in the ward.

Questionnaires: The HCWs’ profession was recorded (medical/paramedic). HCWs were asked to self-report the frequency they performed ABHR at four different times:

- before contact with the patient/after contact with the patient,
- after contact with the patient's close environment and
- before carrying out an invasive measure (e.g., catheter insertion, intubation, injection, urinary catheterisation, incision, etc...).

Answers were on a scale from 0 (never) to 10 (always).

Patients were asked about

1. their age group,
2. how often they observed a HCW performing ABHR before performing a care and measured it on a scale of 0 (never) to 10 (always),
3. if they received information about when to perform ABHR (yes or no).

### Study population

The study included all data from healthcare facilities from 6 June 2019 to 11 November 2023. Two stable cohorts were formed. The first cohort is termed “HCWs Pulpe’Friction cohort”. The second cohort included the wards which interviewed patients during two consecutively audits. This cohort is termed “Patients Pulpe’Friction cohort”.

All wards with at least 30 days between two successive audits were included in the cohorts, in order to give them enough time to perform an intervention. Wards with less than two participants (HCWs or patients) during an audit were excluded.

### Judgement criteria

In the HCWs Pulpe’Friction cohort, to evaluate whether there was an improvement in declared ABHR between two audits, four variables were used: frequency of ABHR before contact with the patient, after contact with the patient, after contact with the patient's close environment, and finally before carrying out an invasive measure (continuous variables).

In the Patients Pulpe’Friction cohort, to evaluate whether there was an improvement in observed HCWs’ ABHR between two audits, the variable “observed frequency of ABHR performed by a HCW before a care measure“ was used (continuous variable). To evaluate whether there was an improvement in the information the patients received about when to perform hand hygiene, the variable “I received information about when to perform hand hygiene” was used (categorical variable).

### Statistical analysis

Descriptive statistics were expressed as percentages for categorical variables and as means with standard deviations and medians with interquartile ranges for continuous variables. Temporal distribution and the time elapsed between the two audits were calculated. The number of HCWs and patients per ward was assessed. The proportion of paramedics in both audits was estimated. The distribution of patient’s age groups was assessed for both audits. 

Quantitative data from HCWs and patients were pooled to obtain mean scores per ward for the questions of interest, scored on a scale from 0 to 10. These mean scores were then used to calculate a global mean score for each audit. As data reported by HCWs and patients in the same ward cannot be considered independent, a Wilcoxon signed-rank test was conducted to evaluate whether there was an improvement in the average distribution of incidence of ABHR between the first and second audit based on clinical situations. A chi-squared test was performed for the qualitative variables. 

Statistical analysis was performed using R Studio^©^ version 2023.12.1+402. All analyses were based on two-sided p-values, with p<0.05 considered statistically significant.

### Ethics approval and consent to participate

Data were anonymized, and after being informed, participants consented to the use of their data. Database management was approved by the ethics committee of the Guadeloupe University Hospital (reference number A11-20-02-21), in accordance with the General Data Protection Regulation of the EU, the French National Commission for Data Protection (CNIL), and French regulations.

## Results

### HCWs Pulpe’Friction cohort

A total of 416 wards were included. The first audit was mainly carried out in 2019, 2021 and 2022 (32.0%, 26.7%, and 32.7%, respectively) (Table 1 (Tab. 1)). More than half of the second audits were carried out in 2023.

The time elapsed between the two audits ranged from 30 to 1,541 days (with a mean of 575 days and a median of 372 days). 

These 416 wards belonged to 130 health facilities in 16 French territories: 12 on the French mainland and 4 in overseas territories and communities (Guadeloupe, Mayotte, Réunion and New Caledonia). Medical, rehabilitation, surgical, and psychiatric wards were the four most represented specialties (28%, 19%, 17% and 11% of all wards respectively) (Table 2 (Tab. 2)). The results did not differ by facility or ward type. 

In the 416 wards, 10,904 interviews of HCWs were collected. During the first audit, 5,521 HCWs were interviewed, and during the second audit, 5,383 HCWs were interviewed. During the first audit, the mean number of HCWs per ward was 13, with a median of 11 and a range of 2 to 136. During the second audit, the mean number of HCWs per ward was also 13, with a median of 10 and a range of 2 to 106. The proportion of paramedics differed between the two audits, with 90% of HCWs who were paramedics in the first audit were being compared to 92% in the second audit (p<0.01). 

Univariate analysis showed an improvement in the global mean scores for declared “frequency of ABHR before contact with the patient” (p-value<0.01), “frequency of hand hygiene after contact with the patient” (p-value<0.01) and “frequency of hand hygiene after contact with the patient’s immediate environment” (p-value<0.01). There was no improvement in ABHR before carrying out an invasive gesture (p-value=0.20) (Table 3 (Tab. 3)).

### Patients Pulpe’Friction cohort

A total of 139 wards were included. The first audits were mainly carried out in 2019, 2021 and 2022 (26.6%, 20.1% and 43.2% respectively) (Table 4). More than half of the second audits were carried out in 2023.

The time elapsed between the two audits ranged from 35 to 1,541 days (with a mean of 522 days and a median of 350 days). 

These 139 wards belonged to 62 healthcare facilities in 15 French territories: 13 in mainland France and 2 overseas territories (Guadeloupe, Réunion). Medical, rehabilitation, surgical and haemodialysis wards were the four most represented specialties (32%, 28%, 17% and 8% of all wards respectively). The results did not differ according to facility. 

**Figure 1:**
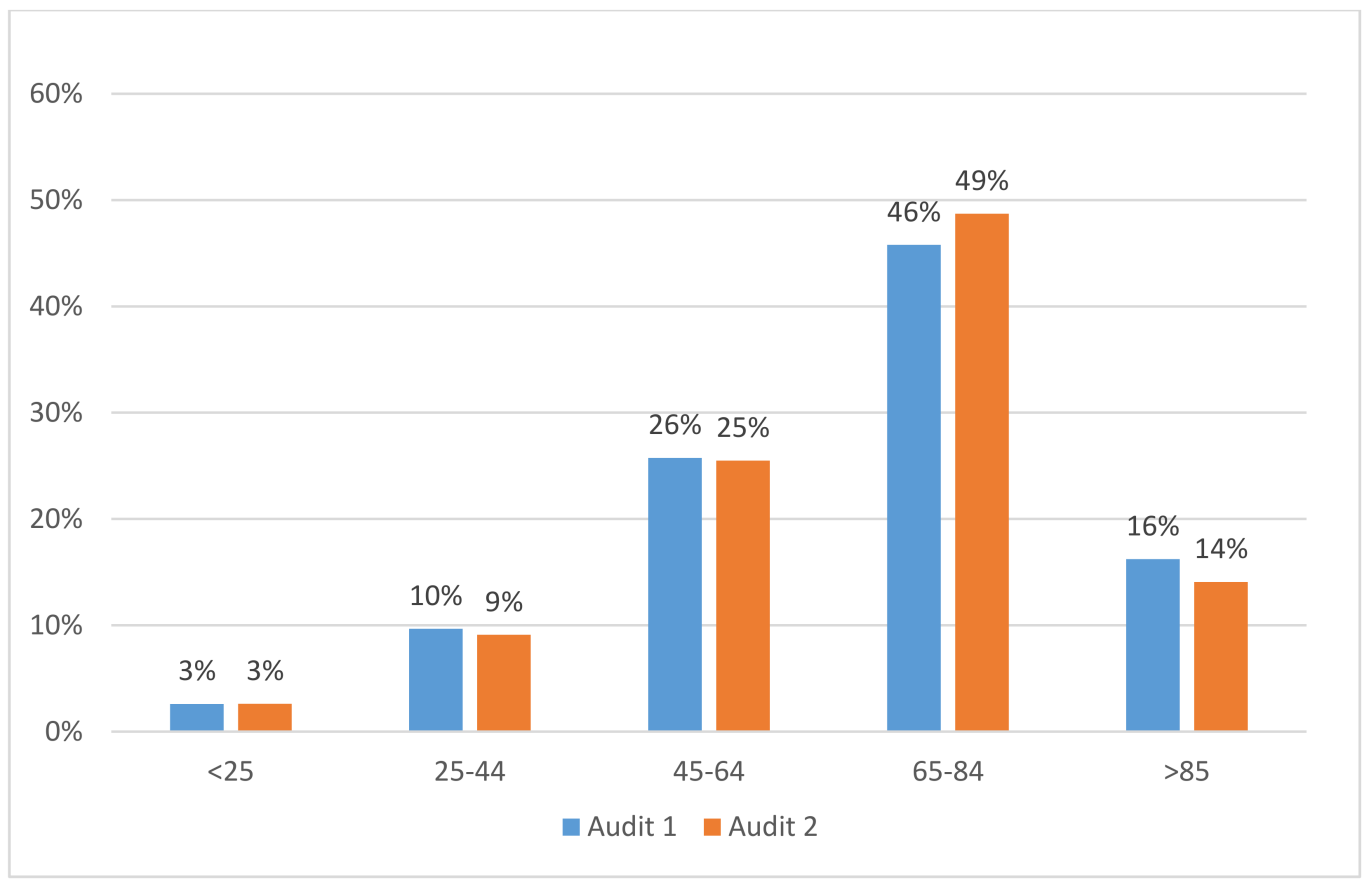
Patient’s distribution (%) by age group (in years) according to the audit in the Patients Pulpe’Friction cohort

In the 139 wards, 3,816 patients responded to the questionnaires. During the first audit, 2,090 patients responded. During the second audit, 1,726 patients responded. During the first audit, the mean number of patients per ward was 15, with a median of 11 and a range of 2 to 100. During the second audit, the mean number of patients per ward was 12, with a median of 8 and a range of 2 to 72. Almost 50% of patients in the two audits were aged between 65 and 84 years (Figure 1 (Fig. 1)). 

In the univariate analysis, there was no improvement in the global mean score for “how often patients observed a HCW performing ABHR before contact with them” (p-value=0.40). 

Patients were significantly better informed about when to perform hand hygiene during the second audit (p<0.01) (Table 5 (Tab. 4)). 

## Discussion

In the wards in which two successive audits were realized, a significant improvement in declared HCWs’ alcohol hand rub practices before and after contact with the patient, and after contact with the patient's immediate environment was observed. In addition, significantly more patients declared they were informed about when to perform hand hygiene during the second audit. 

A significant improvement in HCWs’ declared ABHR suggests that the wards may have implemented actions to improve ABHR in these situations. The second audit measured a positive impact of these actions. Although a discrepancy may exist between self-reported and actual hand hygiene practices among HCWs [10], [11] the significant improvement nevertheless indicates that the intervention worked to enhance good practices. However, there was no improvement of the frequency of ABHR before carrying out an invasive measure; this result can be explained by the fact that the frequency of ABHR was already very high for this situation. 

Several authors used observational measures of ABHR to evaluate their impact on hand hygiene campaigns [12], [13], [14]. The advantage of pulpe’friction interviews is that they are very fast and easy to perform, which proved to be useful to measure an impact. Moreover, interviews create a positive culture of communication by identifying barriers to and facilitators of good compliance [15].

There was no significant improvement in the frequency of ABHR performed by HCWs and observed by the patients before a care measure was performed. However, because this survey did not include questions on whether the 139 wards which performed these patient audits implemented actions between the two audits to promote HCWs’ hand hygiene before touching a patient, it is difficult to interpret this result. 

As the patients were significantly better informed in the second audit about when to perform hand hygiene, these results suggest that the wards might have worked on improving informating the patients. A previous study showed that patients’ observations of alcohol hand rub were correlated with HCWs’ reported practices [7]. Furthermore, the involvement of patients and their family in improving hand hygiene is an ongoing area of research [16]. Our results demonstrate that a quick and simple interview of the patients can be used to evaluate the impact of collective actions. 

The strength of this study lay in its large sample, with results from healthcare facilities in almost all French regions, which reduced selection bias. Investigators were trained to ensure the honesty of the responses and limit interpretation bias. A delay of thirty days between two successive audits was chosen to allow facilities sufficient time to implement new actions to improve ABHR. 

## Limitations

The study has some limitations. Only reported practices were collected and observations of practices were not performed. Besides, the actions which were implemented by the wards between two audits were unknown.

## Conclusion

The wards in which two audits were performed significantly improved their reported practices. This study showed the pulpe’friction method not only offers a way to evaluate collective hand hygiene and ways to improve, it also offers a simple way to evaluate the impact of actions implemented in a ward.

## Notes

**The authors thank the health care workers and the infection control teams who performed the Pulpe’fricton evaluations.**

### Authors’ ORCIDs

- Venier AG: 0000-0002-5077-9820
- Parneix P: 0000-0002-4157-9616
- Nasso R: 0009-0003-5400-0786
- Velardo F: 0000-0002-9293-346X

### Ethical approval 

The database management was approved by the Ethics Committee of the Guadeloupe University Hospital (reference number A11-20-02-21), in accordance with the General Data Protection Regulation of the EU, the French National Commission for Data Protection (CNIL), and French regulations.

### Funding

None

### Competing interests

The authors declare that they have no competing interests.

## Figures and Tables

**Table 1:**
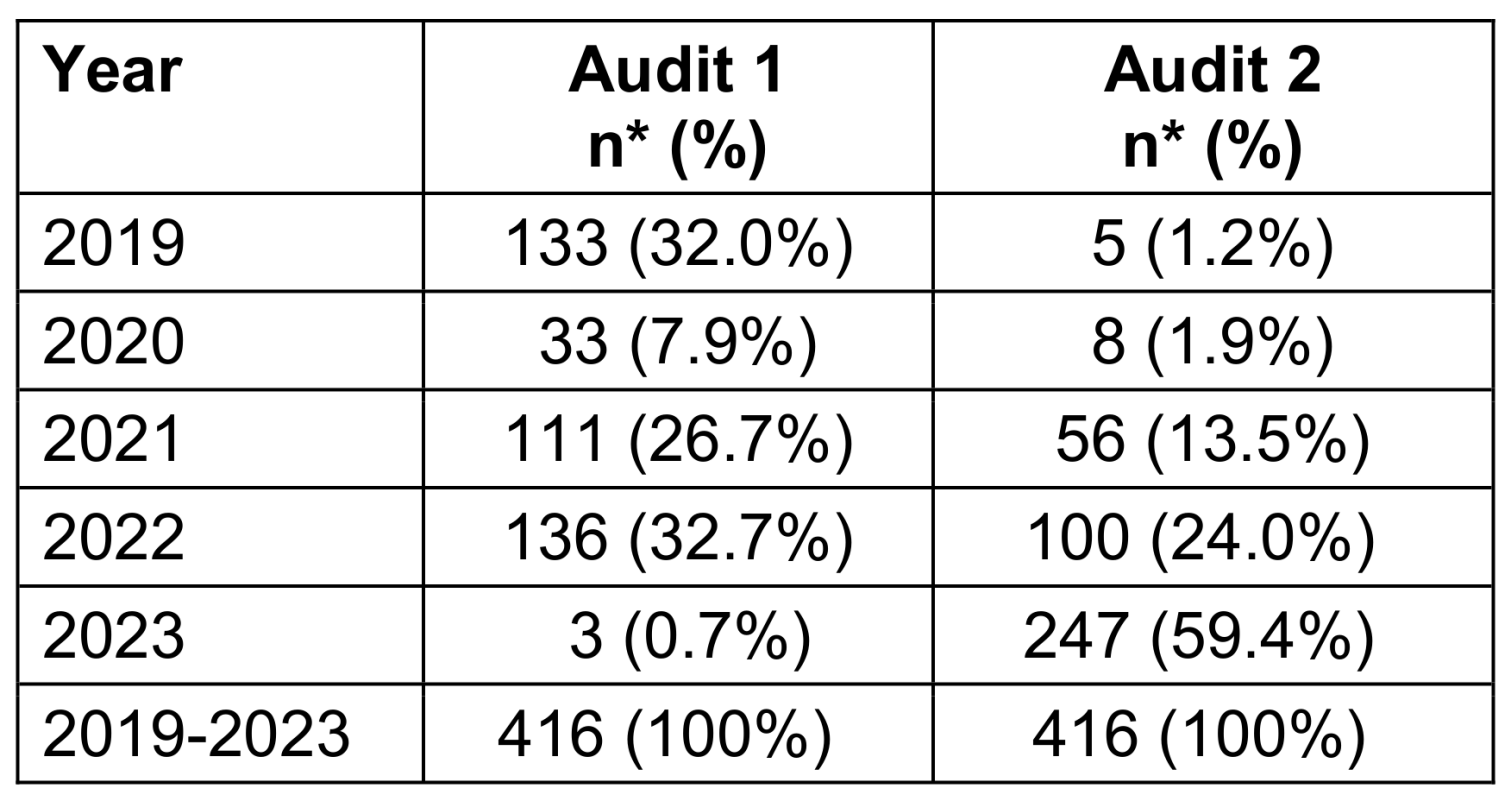
Distribution of wards according the year and the audit in the HCWs Pulpe’Friction cohort

**Table 2:**
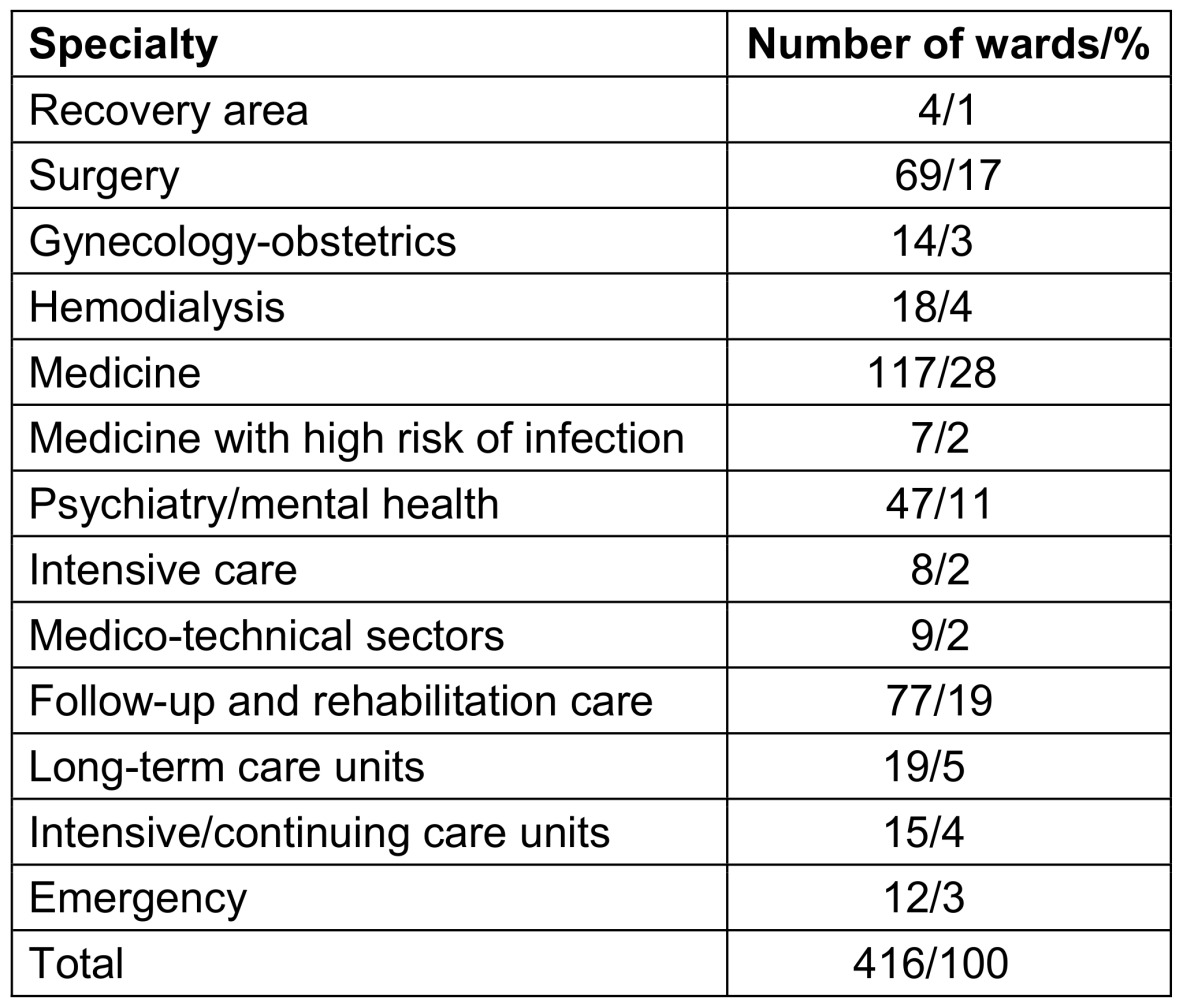
Distribution of the specialty in the HCWs Pulpe’Friction cohort

**Table 3:**
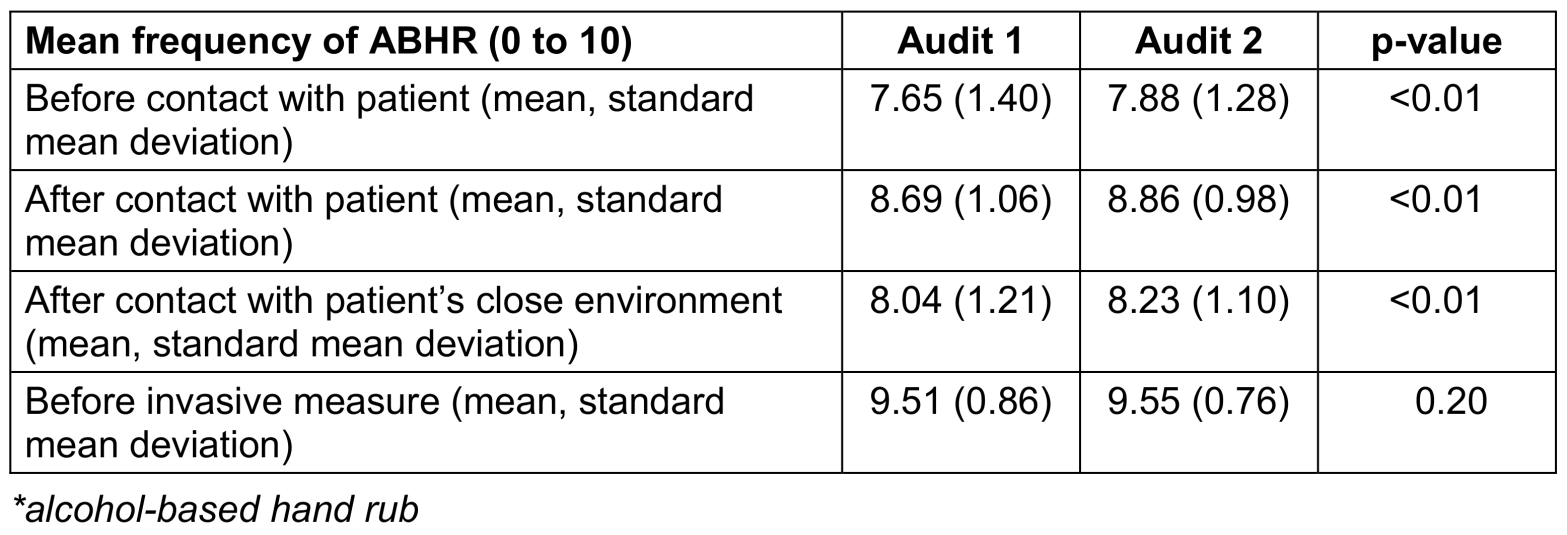
Comparison of the average frequency of self-reported ABHR* practices by health care workers in different situations in the HCWs Pulpe’friction cohort

**Table 4:**
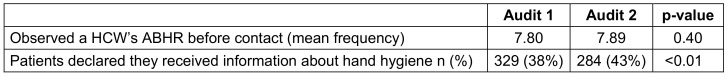
Table 5: Changes in the mean frequency of ABHR performed by HCWs and observed by patients and changes in the number and proportion of patients who declared they received information about hand hygiene in the Patients Pulpe’friction cohort
